# What Happens When Threading is Suppressed in Blends of Ring and Linear Polymers?

**DOI:** 10.3390/polym8120409

**Published:** 2016-11-25

**Authors:** Benjamin Crysup, Sachin Shanbhag

**Affiliations:** Department of Scientific Computing, Florida State University, Tallahassee, FL 32306, USA; brc13c@my.fsu.edu

**Keywords:** ring polymer, cyclic polymer, diffusion, probe diffusion, polymer blend, Monte Carlo simulation

## Abstract

Self-diffusivity of a large tracer ring polymer, $D_{r}$, immersed in a matrix of linear polymers with $N_{l}$ monomers each shows unusual length dependence. $D_{r}$ initially increases, and then decreases with increasing $N_{l}$. To understand the relationship between the nonmonotonic variation in $D_{r}$ and threading by matrix chains, we perform equilibrium Monte Carlo simulations of ring-linear blends in which the uncrossability of ring and linear polymer contours is switched on (non-crossing), or artificially turned off (crossing). The $D_{r}\approx 6.2\times 10^{-7}N_{l}^{2/3}$ obtained from the crossing simulations, provides an upper bound for the $D_{r}$ obtained for the regular, non-crossing simulations. The center-of-mass mean-squared displacement ($g_{3}\left(t\right)$) curves for the crossing simulations are consistent with the Rouse model; we find $g_{3}\left(t\right)=6D_{r}t$. Analysis of the polymer structure indicates that the smaller matrix chains are able to infiltrate the space occupied by the ring probe more effectively, which is dynamically manifested as a larger frictional drag per ring monomer.

## 1. Introduction

Over the past decade, interest in the structure and dynamics of ring polymers (RP) has exploded due to progress in synthesis [1,2,3,4,5], separation [6,7,8,9], and imaging [10,11,12,13]. These advances have allowed us to either produce sufficient quantities of “uncontaminated” RPs, or extract useful information with small samples. Semiflexible polymers like polystyrene with $C_{\infty }\approx 9.6$ in dilute solutions [14], and DNA, which has a Kuhn length of ≈100 nm, have played a vital role in this renaissance.

Uncontaminated and unknotted RPs are difficult to produce and isolate, but crucial; even a modest amount of contamination by linear polymers (LPs), often by-products of prior reactions, can drastically slow down the overall dynamics. For example, Kapnistos et al. found that intentional contamination of cyclic polystyrene rings (160 kDa) with less than 1% linear chains was sufficient to dramatically retard the linear viscoelastic response [15]. Robertson-Anderson and coworkers used fluorescence microscopy to study the dynamics of probe circular DNA, in different matrices, and found significant topological effects [16,17,18]. Recently, the use of RPs as probes of LP melts via neutron spin echo spectroscopy has been been pioneered, in which perturbation of the internal dynamics of the RP is used to glean insights about the matrix it is embedded in [19,20].

As a result of these findings, ring-linear blends (RLBs) have emerged as an important subject of scientific investigation in their own right. In this paper, we focus primarily on RLBs in which the concentration of the RP is small enough to regard these systems as ring probes diffusing in a LP matrix. In such probe or tracer RP systems, for sufficiently large molecular weight constituents, threading of RPs by LPs is implicated in arrested dynamics. This was first recognized in polystyrene tracer diffusion studies in the 1980s [21,22,23], and reestablished more recently with DNA tracer diffusion studies [16]. Computer simulations of flexible and semiflexible RLBs have yielded valuable insights into the threading phenomenon [24,25,26,27,28,29,30,31]. However, the overwhelming majority of these computational studies have focused on symmetric RLBs, in which the molecular weights of the RP ($N_{r}$) and the LP ($N_{l}$) in the blend are equal. A smaller number of computational studies have been reported on asymmetric RLBs in which $N_{r}\neq N_{l}$ [20,32,33,34].

**Figure 1:**
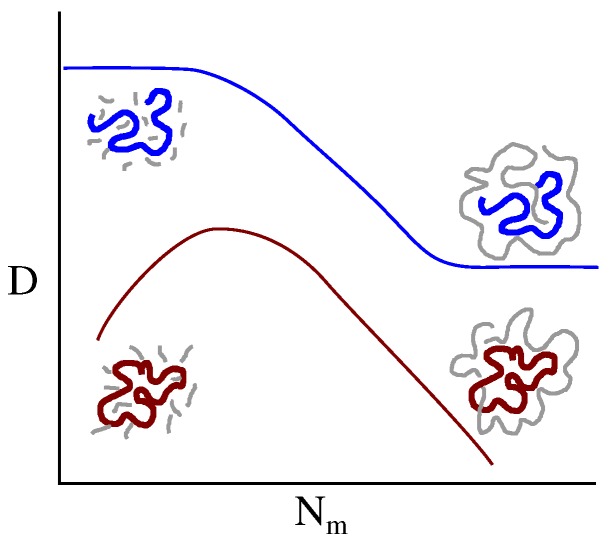
Diffusion of linear (blue) and ring (red) probes in linear matrices (gray). The diffusivity of the linear probe decreases monotonically as the molecular weight of the matrix polymers increases, before reaching a plateau. On the other hand, the diffusivity of ring probes varies non-monotonically. At large $N_{m}$ it decreases without reaching a plateau.

Recently, we described simulations of tracer ring and linear molecules immersed in a matrix of LPs [34]. The molecular weight of the tracer was held fixed, while the length of the matrix LPs ($N_{m}$) was increased from below the entanglement molecular weight $N_{e}$, to 10 $N_{e}$ (see Figure 1). The diffusivity of the linear probe decreased monotonically with $N_{m}$, as anticipated by theory, and observed in experiments and other simulations of binary linear blends [35,36,37,38,39,40,41,42,43,44,45,46]. However, RPs exhibited a surprising non-monotonic variation of the diffusivity with $N_{m}$. Initially the diffusivity of the ring probe $D_{r}$ increased with $N_{m}$, reached a maximum, and then decreased with increasing $N_{m}$, presumably due to ring-linear threading. The behavior of $D_{r}$ for small $N_{m}$ is puzzling, especially since the diffusivity of the matrix chains increases monotonically as $N_{m}$ decreases. Such non-monotonocity in tracer diffusivity is extremely unusual in equilibrium polymeric systems. In binary particle mixtures, similar behavior can be observed only under non-equilibrium conditions with either driven particles, or active media [47,48,49]. Based on available primitive path analysis, we speculated that the non-monotonic behavior probably arose from a competition between the number of ring-linear threading events, and their persistence [34]. As $N_{m}$ decreases, the number of threadings increase, while their persistence—determined by the diffusion timescale of the linear chains in the matrix—decreases.

In this paper, we re-examine this speculation more carefully, by conducting fresh simulations in which threading between RPs and LPs is artificially suppressed, by letting ring and linear contours cross each other. An advantage of simulations is that we can carry out such “unnatural experiments”, which would be hard or impossible to perform in experiments. They are performed with the intention of isolating the effect of particular interactions, while leaving other interactions untouched.

## 2. Materials and Methods

We use the bond-fluctuation model (BFM) [50], which is a lattice Monte Carlo method, to simulate the RLBs. In the past, we have successfully used the BFM to study RLBs [51,52,53,54], due to its ability to efficiently explore long-time scales, and relatively large system sizes. In the BFM, the $C_{\infty }\approx 1.2$ at the typical melt density, compared to $C_{\infty }=1.74$ in the Kremer-Grest off-lattice model [50]. Although the polymer represented in the BFM is flexible, it has been successfully mapped to solutions of dsDNA [18], by matching the number of Kuhn segments.

### 2.1. Bond-Fluctuation Model

In the BFM, 3D space is resolved into simple cubic grid of size L×L×L with periodic boundary conditions. We place $n_{r}$ non-concatenated RPs, each comprising $N_{r}$ beads or monomers, into a matrix containing $n_{l}$ LPs, each comprising $N_{l}$ monomers. To reproduce melt-like behavior, the total density of occupied lattice sites is maintained at $\rho =\left(n_{r}N_{r}+n_{l}N_{l}\right)/L^{3}=0.5$.

Trial moves, in which a randomly selected monomer is displaced to one of its 26 neighboring sites, are attempted. A trial move is accepted if three constraints are satisfied [50]:

1. *Excluded Volume*—the target lattice site is empty and available,
2. *Finite Extensibility*—none of the bond-lengths stretch beyond $\sqrt{3}$, and
3. *Chain Uncrossability*—mid-points of bonds do not intersect.

One Monte Carlo Step (MCS) corresponds to $n_{r}N_{r}+n_{l}N_{l}$ trial moves. The units of distance and time are lattice spacing, and MCS, respectively. The protocol for equilibrating a RLB has been described previously [51,52,53,55]. We monitor the decorrelation of the end-to-end vector and the vector connecting beads 1 and N/2, for LPs and RPs, respectively. Equilibration is terminated once the correlation falls below the threshold value of 0.05. This brute-force protocol yields the correct internal bead distance distribution for situations where it is analytically known.

If the uncrossability constraint is relaxed, chains are allowed to cross and pass through each other. They still have to obey the excluded volume and finite extensibility conditions. Such simulations have been previously peformed on pure LP [50] and RP melts [56]. In this paper, we describe two types of simulations; in the regular or “non-crossing” (NC) simulations, the chain uncrossability constraint is strictly enforced. In the “crossing” (CX) simulations, the chain uncrossability constraint is selectively relaxed only for ring-linear interactions. Therefore, contours of RPs and LPs are allowed to pass through each other. However, uncrossability is strictly enforced for ring-ring, and linear-linear interactions. Thus, these CX simulations, allow us to isolate and explore the dynamical consequences of suppressing threading between RPs and LPs, while leaving other interactions untouched. The acceptance ratio of the Monte Carlo moves for large polymers is found to be independent of polymer architecture; for NC simulations it is about 0.18, while it is about 10% higher for CX simulations.

### 2.2. Self-Diffusion Coefficient

After equilibration, we perform production runs for simulation time $\tau_{\mathrm{sim}}$. We monitor the mean-squared displacement (MSD) of the center of mass,

(1) $$g_{3}\left(t\right)=\langle {\left(r_{C}\left(t\right)-r_{C}\left(0\right)\right)}^{2}\rangle ,$$

where $r_{C}$ denotes the center-of-mass, and 〈·〉 denotes an average over all polymers and time-intervals *t*. The self-diffusivity of the polymers can be obtained from $g_{3}\left(t\right)$, using the Einstein formula:

(2) $$D=\lim_{t\to \infty }\frac{g_{3}\left(t\right)}{6t}.$$

The simulation time was chosen to be long enough to ensure that the polymers had diffused, on average, at least five times their radius of gyration. We used statistical bootstrap [57,58] to infer confidence intervals for the estimated self-diffusivities.

### 2.3. Systems Studied

We studied two sets of RLBs, (i) symmetric; and (ii) asymmetric. The details of these systems are presented in Table 1. In the bond-fluctuation model, at ρ=0.5, the average number of monomers per entanglement segment is $N_{e}\approx 30$ [59,60,61].

- In *symmetric* blends, $N_{r}$ = $N_{l}$ = 300 was held fixed, while the linear fraction $\phi_{l}$ = $n_{l}N_{l}/\left(n_{l}N_{l}+n_{r}N_{r}\right)$ was varied between 0 and 1. Note that for $\phi_{l}$ = 0 (pure rings) and $\phi_{l}$=1 (pure linears), the crossing simulations and the non-crossing simulations were identical, since there are no ring-linear interactions to suppress in these pure systems.
- In *asymmetric* blends, the concentration of the matrix LPs $\phi_{l}=0.9$, and the number of RP monomers $N_{r}=300$, were held fixed, while the number of LP monomers was varied between $N_{l}=10-300$. To avoid ring-ring interactions in these probe systems, the concentration of the ring polymers was kept about 10 times lower than the overlap concentration [34].

## 3. Results

In the following, we discuss the static and dynamic properties of the symmetric and asymmetric blends. The results of all the NC simulations have been previously reported, including the size [51], entanglement structure [53], free energy [54], and self-diffusion [55] of the symmetric $N_{r}=N_{l}=300$ blends, and the size and diffusivity of the asymmetric probe ring blends [33,34].

### 3.1. Symmetric Blends

When the non-crossing constraint is relaxed in the symmetric RLBs, the change in static properties is barely perceptible, while the self-diffusivity profiles change considerably. Symmetric blends provide a baseline from which the results of the more interesting asymmetric blends can be analyzed.

#### 3.1.1. Statics

**Figure 2:**
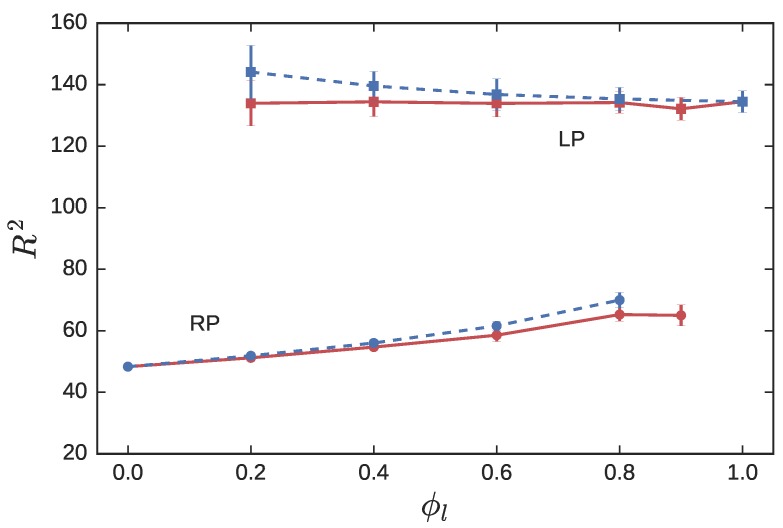
The squared radius of gyration of RPs (circles) and LPs (squares) in a symmetric blend with $N_{l}=N_{r}=300$, as a function of the linear fraction. Solid red lines represent NC simulations, while blue dashed lines represent CX simulations.

Figure 2 plots the squared radius of gyration, $R^{2}$, of the RPs and LPs in the blend. In the NC simulations, the mean radius of gyration of the LPs $R_{l}^{\mathrm{NC}}\approx 11.6$. Superscripts “NC” and “CX” are used to distinguish properties extracted from non-crossing and crossing simulations, and the subscripts “*r*” and “*l*” are used to represent RPs and LPs, respectively. In the crossing simulations, the mean $R_{l}^{\mathrm{CX}}$ increases from 11.6±0.2 to 12.0±0.4, as $\phi_{l}$ decreases from 1.0 to 0.2. In previous comparisons of NC and CX simulations of pure LPs [50,62], no significant differences in polymer size were observed. However, in those simulations, the LPs were allowed to pass through each other, unlike our simulations in which LP-LP crossings are prohibited, and only LP-RP crossings are allowed.

RPs in pure melts adopt highly compact conformations due to the non-catenation constraint between neighboring rings. Brown et al. [56] performed CX simulations of pure ring melts, in which the non-catenation constraint was relaxed by allowing RP-RP crossings. This caused the $N_{r}=300$ pure ring melts to swell from $R_{r}^{\mathrm{NC}}=7.0$ to $R_{r}^{\mathrm{CX}}=8.3$. In both our CX and NC calculations, RP-RP crossings are not permitted. However, a similar effect is observed by contaminating a pure ring melt with increasing levels of LPs. As the linear fraction $\phi_{l}$ increases (Figure 2), LPs weaken the strength of the non-catenation constraint due to dilution of ring-ring interactions, causing the RP to expand. In RLBs that are LP-rich (large $\phi_{l}$), the RPs adopt conformations consistent with Gaussian rings [51]. In the NC simulations, the size of the RP increases from 7.0±0.1 to 8.1±0.1, as $\phi_{l}$ increases from 0 to 0.9. In the CX simulations, the RP size increases from 7.0±0.1 to 8.3±0.1.

The small enhancement in the LP size in RP-rich blends, and in RP size in LP-rich blends for the CX simulations relative to the corresponding NC simulations can be undestood in terms of a local “solvation effect”. For example, a LP surrounded predominantly by “crossable” RPs in RP-rich environments feels that it is in a (partially) good solvent, prompting it to swell. The degree of relative swelling depends on the concentration of the opposite species in the blend.

#### 3.1.2. Dynamics

**Figure 3:**
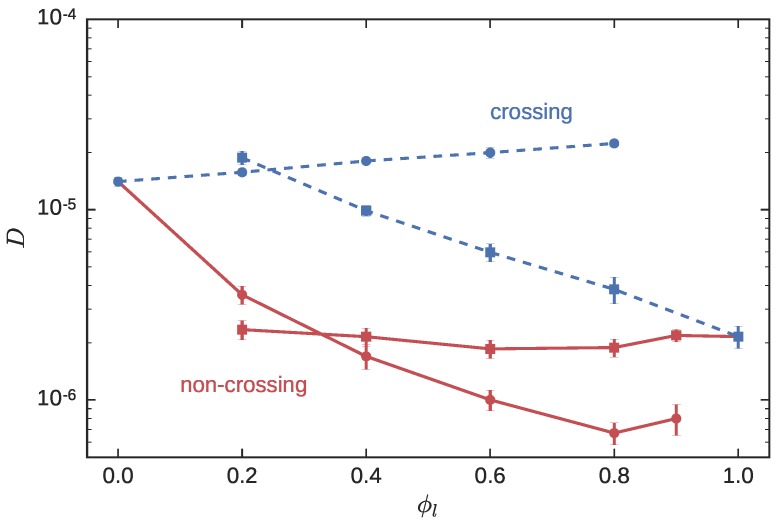
The diffusivity of RPs (circles) and LPs (squares) in a symmetric blend with $N_{l}=N_{r}=300$, as a function of the linear fraction. Red solid lines represent NC simulations, while blue dashed lines represent CX simulations.

Figure 3 depicts the diffusivity of the LPs and RPs in the blend for NC and CX simulations. In the NC simulations, diffusivity of the LP, $D_{l}\approx$ constant, although there is a shallow minima at large $\phi_{l}$, which is also observed experimentally [18]. On the other hand, the diffusivity of the RP decreases precipitously from the pure melt ($\phi_{l}=0$) as the linear fraction increases. This is due to threading of the RPs by the LPs; the RPs are pinned down by LPs, and are effectively immobilized on the diffusion timescale ($\tau_{l}∼R_{l}^{2}/D_{l}$) of the threading LPs.

When ring-linear threading is artifically switched off in the CX simulations, the RP is no longer constrained, and its diffusivity actually *increases* from $D_{r}^{\mathrm{CX}}=1.4\times 10^{-5}$ to $2.3\times 10^{-5}$ as $\phi_{l}$ increases from 0 to 0.8. The increase in linear in $\phi_{l}$ and is well-described by the relation, $D_{r}^{\mathrm{CX}}\left(\phi_{l}\right)=1.4\times 10^{-5}\left(1+0.8\phi_{l}\right)$. The increase in $D_{r}^{\mathrm{CX}}$ with increasing $\phi_{l}$ is due to the replacement of uncrossable ring neighbors with LPs, which a RP can cut through. This can again be thought of as a manifestation of the solvation effect, which was responsible for a small increase in $R_{r}$ (Figure 2) with $\phi_{l}$. For the LPs, a corresponding solvation effect is manifested by the increase in $D_{l}$ from $2.2\times 10^{-6}$ to $1.9\times 10^{-5}$ as $\phi_{l}$ decreases from 1.0 to 0.2. The data is well-described by $D_{l}^{\mathrm{CX}}\approx 2.2\times 10^{-6}\exp\left(2.6\left(1-\phi_{l}\right)\right)$. As we move away from a LP-rich to a RP-rich environment, the fraction of the medium offering topological resistance to a LP goes down.

In the CX simulations, the RP or the LP sees the opposite species as a spatial correlated high-density solvent, offering some resistance. In other words, the presence of LPs as $\phi_{l}\to 1.0$ slows RPs below their mobility in the absence of LPs. This is evident from the CX computations of Brown et al. [56] referenced earlier. In their work, the found that the diffusivity of $N_{r}=300$ RPs in a pure ring melt increased from $1.3\times 10^{-5}$ to $4.1\times 10^{-5}$, when the RP-RP crossing was allowed. However, this diffusivity is still smaller than the diffusivity of single isolated non-crossing ($D_{r}=1.4\times 10^{-4}$) or crossing ($D_{r}=1.6\times 10^{-4}$) RPs.

### 3.2. Ring Probes in Linear Matrix

The results of the static and dynamic properties of symmetric RLBs seem intuitive. We now turn our attention to asymmetric blends, with $N_{r}=300$, in LP-rich matrices comprised of varying molecular weights $N_{l}$.

#### 3.2.1. Statics

**Figure 4:**
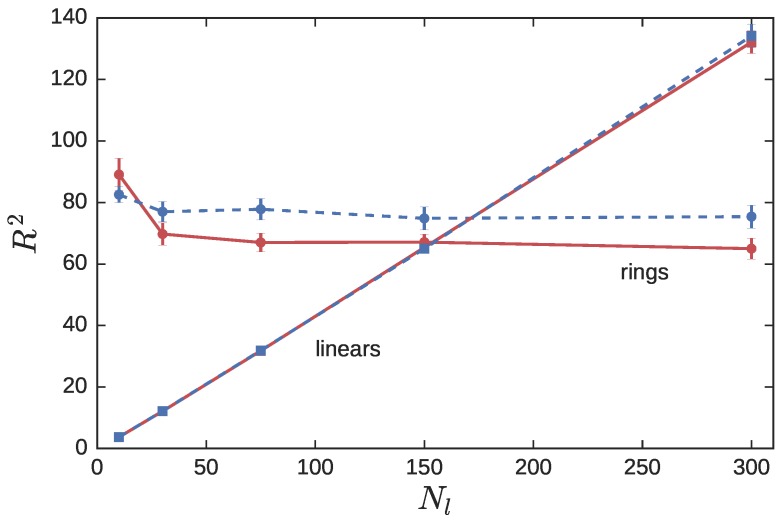
The squared radius of gyration of RPs (circles) and LPs (squares) in asymmetric blends with $N_{r}=300$, and $N_{l}$ varied between 10 and 300. The linear fraction $\phi_{l}$ is held fixed at 0.9. Red symbols connected with solid lines represent non-crossing simulations, while blue symbols and dashed lines represent crossing simulations.

The results of the NC simulations have been reported in a recent publication [34]. To summarize, the radius of gyration of the LPs $R_{l}^{\mathrm{NC}}∼N_{l}^{1/2}$, while that of the probe RP is essentially a constant over a large range of matrix molecular weights. As $N_{l}$ falls below $N_{l}^{*}=\sqrt{N_{r}}$, the matrix LPs act as a solvent causing the ring probe to swell by about 10% (Figure 4).

$R_{l}^{\mathrm{CX}}$ of the LPs in the CX simulations essentially overlaps with the $R_{l}^{\mathrm{NC}}$ from the NC simulations. This is anticipated from Figure 2. At $\phi_{l}=0.9$, a LP is essentially surrounded by other non-crossable LPs. The presence of a few crossable probe RPs in the melt induces the LPs to expand; however, their concentration is too low to produce significant observable differences.

On the other hand, the size of the ring probes is noticeably different in the CX and NC simulations. At $\phi_{l}=0.9$, the neighborhood of a RP consists predominantly of crossable LPs, which allow the RP to swell. Indeed the plateau value of $R_{r}^{2}$ for $N_{l}\geq 75$ increases from 65.0±3.4 to 75.4±3.8 as we move from NC to CX calculations (Figure 4). The upturn observed in $R_{r}$ for $N_{l}<N_{l}^{*}$, is muted in the CX simulations due to the weakened impact of the solvation effect.

#### 3.2.2. Dynamics

**Figure 5:**
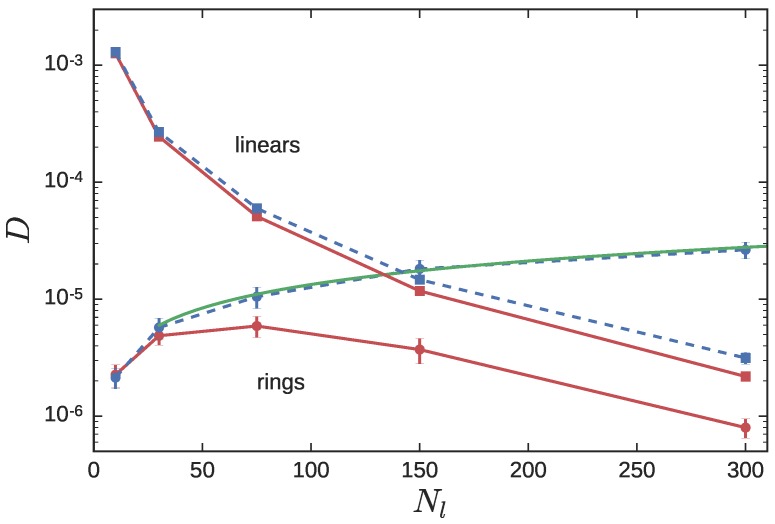
The diffusivity of $N_{r}=300$ probe RPs (circles) in LP (squares) matrix, as $N_{l}$ is varied between 10 and 300. The linear fraction $\phi_{l}$ is held fixed at 0.9. Red symbols connected with solid lines represent NC simulations, while blue symbols and dashed lines represent CX simulations. The solid green line is the fitting function $6.2\times 10^{-7}N_{l}^{2/3}$.

Figure 5 depicts the self-diffusion constant of the CX and NC probe ring systems. The NC simulations were previously reported [34]. The diffusivity of the LPs in the blend $D_{l}$ is a monotonically decreasing function of $N_{l}$. At $\phi_{l}=0.9$, $D_{l}$ tracks the diffusivity of pure LPs, which for $N_{l}>75$, varies as $D_{l}∼N_{l}^{-2.4}$ [34]. The diffusivity of the RP $D_{r}^{\mathrm{NC}}$, on the other hand, exhibits a surprising non-monotonic behavior, which was alluded to previously. Primitive path analysis suggested that for RP probes, there were two regimes: when $N_{l}$ was small, the number of ring-linear entanglements decreased with $N_{l}$, eventually crossing over to a plateau as the size of the LP matrix chains became comparable with that of the RP. It was speculated that the increased degree of entanglement more than offset the effect of the increasing mobility of the matrix chains, in the small $N_{l}$ regime, leading to a non-monotonic variation in $D_{r}$.

The dashed blue lines and symbols in Figure 5 depict $D_{l}$ and $D_{r}$ in the CX simulations. At $\phi_{l}=0.9$, a LP sees a LP-rich environment. Since LP-LP contour crossing events are prohibited, $D_{l}$ in the CX simulations closely tracks the $D_{l}$ observed in the NC simulations. The presence of the crossable RPs causes the $D_{l}^{\mathrm{CX}}>D_{l}^{NC}$, as the LPs are marginally more mobile. This effect is stronger at $N_{l}=300$, than at $N_{l}=10$, and as $N_{l}$ decreases, the enhancement in mobility weakens systematically. When $N_{l}$ falls below the entanglement threshold $N_{e}\approx 30$, the strength of the non-crossing topological constraint is reduced even in the NC simulations, and the difference between the CX and NC simulations becomes less important.

This reduction in the significance of ring-linear threading as $N_{l}$ decreases is also evident in the diffusivity of the RP, $D_{r}$. In Figure 5, the values of $D_{r}$ in both the CX and NC simulations merge at small $N_{l}$, as one would expect. Indeed, the $D_{r}^{\mathrm{CX}}$ in the CX simulations provides an upper-bound for $D_{r}^{NC}$. For $N_{l}<N_{e}$, the LPs do not effectively constrain the RPs. As $N_{l}$ increases to 75 (about $2N_{e}$) and above, threading of the RPs by the matrix chains begins controlling the long-time dynamics of the RP, and the $D_{r}$ in the CX and NC simulations start to diverge. In the NC simulations, the $D_{r}$ decreases as $N_{l}$ increases. In the CX simulations, for $N_{l}\geq 30$ the power-law dependence $D_{r}^{\mathrm{CX}}\approx 6.2\times 10^{-7}N_{l}^{2/3}$ describes the data quite well.

## 4. Discussion

When Shaffer [50] performed BFM simulations with pure LP melts by switching the uncrossability criterion on and off, he found Rouse-like scaling of the LP diffusivity in the crossing simulations $D_{l}^{\mathrm{CX}}=0.0131N_{l}^{-1}$. Furthermore, this diffusivity provided an upper-bound to the diffusivity of the LPs in the NC simulations, $D_{l}^{NC}\leq D_{l}^{\mathrm{CX}}$. This pattern is observed in the results reported in Figure 3 and Figure 5. The CX simulations provide an envelope under which $D^{NC}$ is forced to lie. The key question to address, to completely understand the non-monotonic varition of $D_{r}^{NC}\left(N_{l}\right)$, is “why does $D_{r}^{\mathrm{CX}}$ increase monotonically with $N_{l}$ in Figure 5?”

We saw from Figure 4, that the size of the LP in the CX simulations was relatively unchanged from the NC simulations, while the RP was somewhat more expanded (except at the smallest $N_{l}$ explored). We wanted to examine how this affects the local microenvironment of a RP; in particular, to quantitatively address the question, “how many neighboring polymers infiltrate the space occupied by a ring probe?” Therefore, we considered a probe RP and computed the number of polymers of either species (RP or LP) whose centers-of-mass were contained within the radius of gyration $R_{r}$ of the probe.

**Figure 6:**
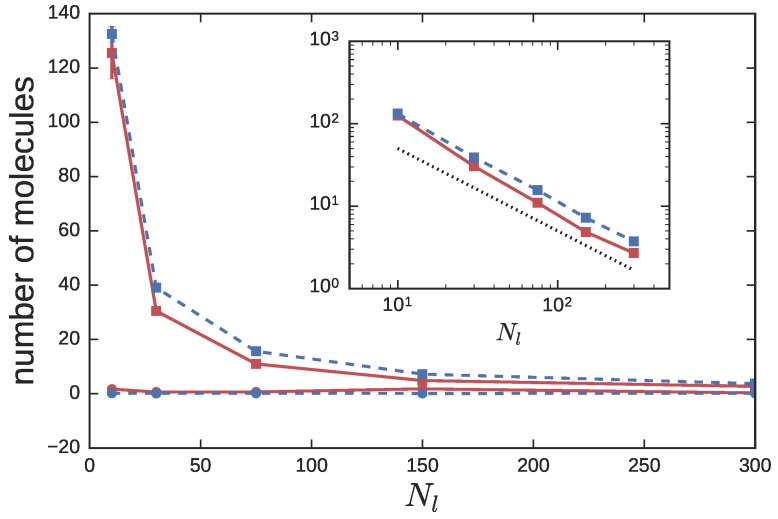
The number of polymers—RPs (circles) and LPs (squares)—contained within one radius of gyration of a probe RP in the asymmetric blend simulations. Red solid lines represent NC simulations, while blue dashed lines represent CX simulations. The inset replots the LP data on a log-log scale. The dotted line is proportional to $N_{l}^{-1}$.

Figure 6 shows that the number of RPs within this region is nearly zero for both the CX and NC simulations. This is expected, since at $\phi_{l}=0.9$, the concentration of the RPs is significantly below its overlap concentration. The number of LPs within this region increases as $N_{l}$ decreases. As $N_{l}$ decreases, the LPs become smaller in size, and are better able to sneak into small voids available within a RP. The inset to the figure shows the number of LPs on a double logarithmic scale. The difference between the CX and NC simulations becomes smaller as $N_{l}$ decreases. For larger $N_{l}$, the number of LPs in the CX simulations lies above that in the NC simulations, presumably due to the larger size of the probe rings in these simulations (see Figure 4).

**Figure 7:**
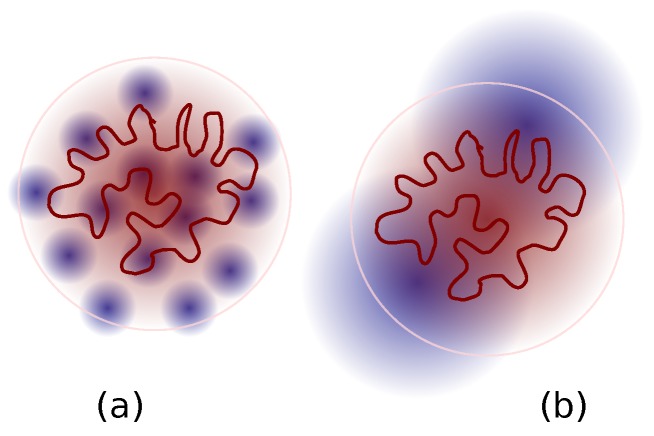
Schematic of infiltration of a ring probe by matrix LPs of (**a**) low; and (**b**) high, molar mass $N_{l}$.

Since the number of LPs within $R_{r}$ (let us label it $n_{l}^{R}$) varies with $N_{l}$ as $n_{l}^{R}∼N_{l}^{-1}$ from the inset to Figure 6, one can conclude that $n_{l}^{R}N_{l}\approx$ constant. This suggests that the number of LP monomers contained within $R_{r}$ of a ring probe, $n_{l}^{R}N_{l}$, is independent of the molecular weight of the LP. In other words, fraction of sites occupied by the LPs in the local microenvironment of a RP is roughly unchanged as a function of $N_{l}$. For the NC simulations, there appears to be a stronger dependence of $n_{l}^{R}$ on $N_{l}$ at smaller $N_{l}$. This observation is consistent with previous primitive path simulations [34], which showed enhanced ring-linear entanglement in this regime. The shorter LPs are more mobile, and better dispersed within the pervaded volume (see Figure 7). They can thus offer better frictional resistance to the internal modes of motion of the RP.

**Figure 8:**
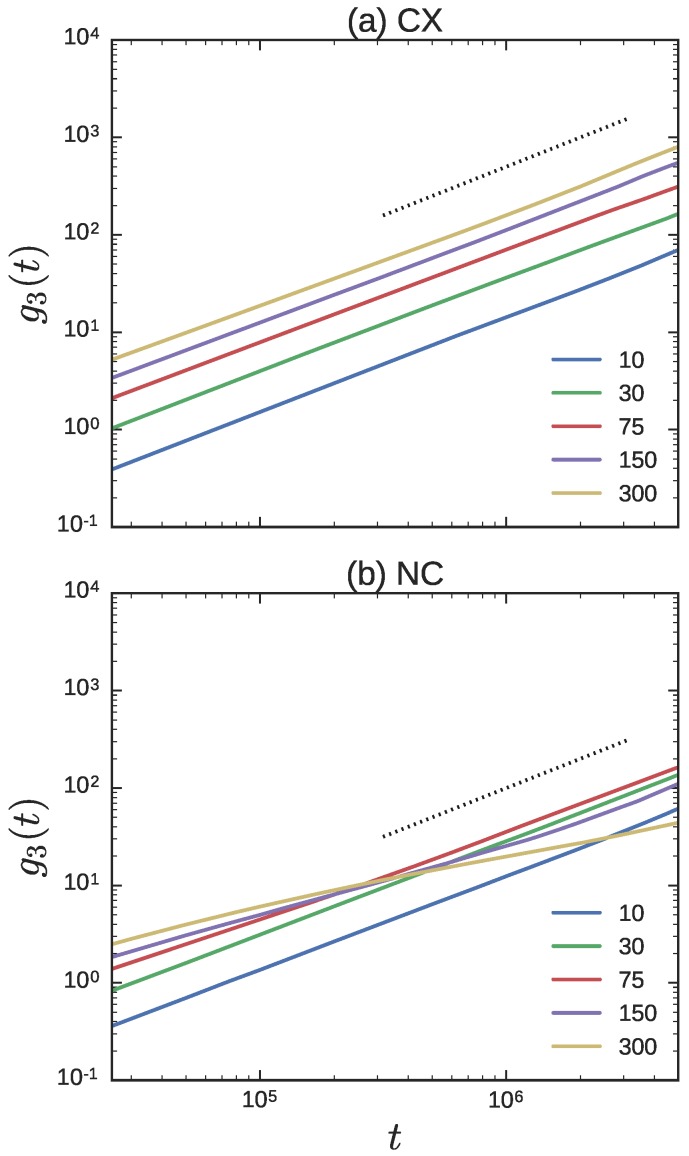
The $g_{3}\left(t\right)$ curves of the probe rings for the (**a**) crossing; and (**b**) non-crossing simulations. Different lines correspond to different $N_{l}$ indicated in the legend. The dotted line in both figures has a slope of one.

Figure 8 depicts the $g_{3}\left(t\right)$ for the crossing and non-crossing ring probes as $N_{l}$ is varied, over small to intermediate timescales. The $g_{3}\left(t\right)$ for the NC RPs are similar to the $g_{3}\left(t\right)$ curves obtained in previous tracer diffusion studies [34]. In tracer diffusion studies of a large LP immersed in a matrix of shorter LPs, the $g_{3}\left(t\right)$ curves of the tracer LP become independent of the matrix molecular weight at short timescales. In constrast, the $g_{3}\left(t\right)$ curves of a tracer or probe RP at short timescales increases with increasing $N_{l}$—a characteristic which is also observed in simulations of pure ring melts of varying molar mass [34]. As $N_{l}$ decreases, the matrix chains are able to infiltrate the space occluded by a ring probe more effectively. Therefore the $g_{3}\left(t\right)$ of the RP feels the effect of the matrix chains at shorter times. At intermediate timescales, the effect of threading becomes visible, especially for the longest $N_{l}$.

Unlike the complicated structure of the $g_{3}\left(t\right)$ curves in the NC simulations, the $g_{3}\left(t\right)$ curves in the CX simulations are simple. The terminal diffusive regime, as attested by the transition to the $t^{1}$ scaling, appears to be attained at smaller timescales. It is clear that the ring probes are faster at all timescales in the $N_{l}=300$ matrix compared to the the $N_{l}=10$ matrix. The $g_{3}\left(t\right)$ curves are effectively parallel to each other, with the diffusion constant reported in Figure 5 serving as the scaling factor. Indeed, this is strongly reminiscent of the $g_{3}\left(t\right)$ curves for RPs expected from Rouse theory [63]. In the Rouse model, $g_{3}\left(t\right)=6D_{r}t$, with $D_{r}=k_{B}T/N_{r}\zeta$, where $k_{B}$ is the Boltzmann constant, *T* is the absolute temperature, $N_{r}$ the number of RP monomers, and *ζ* is the drag per bead.

From Figure 8, the $g_{3}\left(t\right)$ curves for the probe RP depend on $N_{l}$. Since $D_{r}^{\mathrm{CX}}∼N_{l}^{2/3}$ is an increasing function of $N_{l}$, it implies that $\zeta ∼N_{l}^{-2/3}$ is a decreasing function of $N_{l}$. In other words, the effective drag on the beads of the Rouse RP is large when the matrix LPs are small, and are able to enter and disperse into the volume pervaded by the RP more effectively. Dynamics of isolated RPs in a matrix of fixed obstacles at low obstacle density [64] also show qualitatively similar profiles for $g_{3}\left(t\right)$. As the obstacle density is increased from zero, the frictional drag per RP segment increases. The RP is more effectively slowed down, even as the shape of $g_{3}\left(t\right)$ remains essentially unchanged. Beyond a certain obstacle density, the shape of the curve changes.

**Figure 9:**
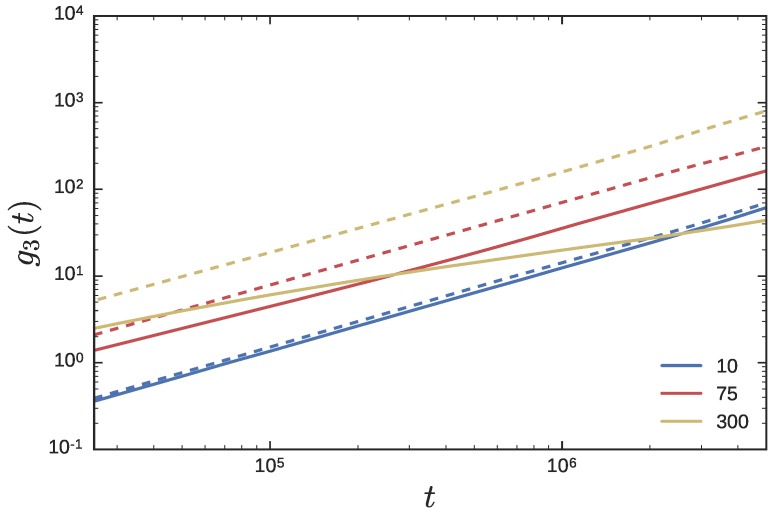
The $g_{3}\left(t\right)$ curves of the probe rings for $N_{l}$ = 10, 75, and 300 for the CX (dashed) and NC simulations (solid) simulations from Figure 8 are plotted together.

Finally, Figure 9 compares the $g_{3}\left(t\right)$ curves between the NC and CX simulations from Figure 8, directly for three different values of $N_{l}$. When the LP is small, $N_{l}\approx 10$, the $g_{3}\left(t\right)$ of the RP probes is nearly the same in the CX and NC simulations. As $N_{l}$ increases to the entanglement threshold and beyond, the $g_{3}\left(t\right)$ of the probes in the CX simulations show greater mobility than in the NC simulations. For $N_{l}=75$ in Figure 9, the $g_{3}\left(t\right)$ of the crossing and non-crossing curves are roughly parallel over the timescales reported. As $N_{l}$ increases further, to say $N_{l}=300$, and the threading by LPs becomes more persistent, the two lines are no longer parallel to each other. The signature of the threading by the LPs is evident in the decreased slope in the $t>10^{5}$ time range.

### Proposed Experiments and Simulations

Although the BFM models a flexible polymer, the provocative results reported in this work have direct implications for semiflexible polymers. First, most practically realizable RP systems are composed of semiflexible polymers; therefore to experimentally validate the BFM observations, we need to figure out the appropriate experiments to perform. Fortunately, the BFM has previously been mapped to solutions of dsDNA polymers. In the BFM, at the densities studied, the relationship between the number of monomers, and the number of Kuhn steps $N_{K}$ is $N_{K}=0.83$ *N*. Using the blob theory, the number of Kuhn steps corresponding to a 45 kbp dsDNA in a 1 mg/mL solution is found to be $N_{K}=263$ [18], which corresponds to N=263/0.83≈317 in the BFM. Thus, the N=300 probe, studied in this and previous work [34], is quite similar in size to a 45 kbp dsDNA in a 1 mg/mL solution. Thus, the *N* = 10–150 matrix polymers correspond approximately to dsDNA of lengths between 1.5–22.5 kbp. Thus experiments in which the diffusivity of a 45 kbp RP is measured in LP matrices whose lengths are varied between 1.5–22.5 kbp at a total concentration of 1 mg/mL will be useful to validate the BFM results.

Furthermore, the effect of stiffness in both the probe and the matrix polymers is an interesting topic to explore, especially using an off-lattice model. The observed non-monotonic effect is due to non-intuitive interplay between the structure and the dynamics of the probe RP. While the semiflexibility of the polymers definitely affects the structure, whether it enhances or subdues the observed non-monotonicity is an open question.

## 5. Summary and Conclusions

Unlike LP probes, the diffusivity of RP probes $D_{r}$ in linear matrices, exhibits an unusual non-monotonic dependence on the matrix molecular weight. For $N_{r}=300$, the diffusivity initially rises for $N_{l}<N_{e}$, reaches maxima around $N_{l}\approx 2$ $N_{e}$, and then decreases with $N_{l}$ due to threading by matrix LPs. Previous work on the entanglement structure suggested that the non-monotonicity in $D_{r}$ could result from a competition between the number of ring-linear threading events and their persistence.

In order to investigate this hypothesis directly, we performed equilibrium Monte Carlo simulations of ring-linear blends using the BFM. In the BFM, the uncrossability criterion, which ensures that contours of polymers do not cut through each other, can be selectively switched off. In our crossing simulations, we turned off the uncrossability condition between RPs and LPs, while keeping it on for ring-ring and linear-linear interactions. This surgically eliminates the role of threading in the CX simulations, thereby allowing us to appreciate its role in the regular NC simulations.

We performed simulations on two sets of systems: symmetric and asymmetric. For the symmetric simulations, the change in the static and dynamics properties in the CX simulations relative to the NC simulations was expected. The size and self-diffusivity both increased as the composition of the opposite species in the blend was increased. The increase in size was marginal, while the increase in self-diffusion coefficient was more dramatic. The changes could be interpreted by a “solvation effect”: the replacement of neighbors by “crossable” polymers of the opposite species allows the polymers to expand, and move faster.

We then explored the size and diffusivity in LP-rich asymmetric blends. The linear fraction was maintained at $\phi_{l}=0.9$, $N_{r}=300$ was held fixed, and $N_{l}$ was varied between 10 and 300. For LPs, there was no significant change in $R^{2}$ or $D_{l}$ between the NC and CX simulations. This was expected, since at $\phi_{l}=0.9$, the environment of a typical LP consists of mostly other LPs. The RP probe expanded slightly due to the solvation effect mentioned above. In the CX simulations, $D_{r}^{\mathrm{CX}}$ increased monotonically with $N_{l}$ and provided and upper-bound for $D_{r}^{NC}$. For $N_{l}\geq 30$, it was found that $D_{r}^{\mathrm{CX}}\approx 6.2\times 10^{-7}N_{l}^{2/3}$.

Investigation of the center-of-mass mean-squared displacement showed that $g_{3}^{\mathrm{CX}}\left(t\right)=6D_{r}^{\mathrm{CX}}t$), consistent with the Rouse model over all the timescales (Figure 8). Analysis of the polymer structure in the CX simulations indicated that the number of LPs that infiltrate the volume occupied by a RP scales as $N_{l}^{-1}$. In the NC simulations, for large $N_{l}$ the number of infiltrating LPs also varied as $N_{l}^{-1}$, while the slope was stronger at shorter $N_{l}$. This is consistent with previously reported primitive path analysis on the NC systems [34], which showed an increase in the topological interactions in this regime. Together, they indicate that the space occupied by the RP is incrementally enriched with matrix polymers as $N_{l}$ decreases as shown in Figure 7. This infiltration is manifested as a larger frictional drag per RP bead in the $g_{3}^{\mathrm{CX}}\left(t\right)$ curves.

## Figures and Tables

**Table 1:** Description of the symmetric ($N_{r}=N_{l}$) and asymmetric blends ($N_{r}\neq N_{l}$) blends simulated in a periodic cubic box with L=60, at a total density of ρ=0.5. Simulation times for the non-crossing (NC) and crossing (CX) simulations are in units of $10^{6}$ Monte Carlo Steps.

$N_{r}$	$N_{l}$	$n_{r}$	$n_{l}$	$\phi_{l}$	$\tau_{\mathrm{sim}}$ (NC)	$\tau_{\mathrm{sim}}$ (CX)
**Symmetric blends**						
300	300	360	0	0.0	30.0	30.0
300	300	288	72	0.2	70.0	20.0
300	300	216	144	0.4	70.0	20.0
300	300	144	216	0.6	80.0	20.0
300	300	72	288	0.8	80.0	20.0
300	300	36	324	0.9	100.0	15.0
300	300	0	360	1.0	40.0	40.0
**Asymmetric blends**						
300	10	36	9,720	0.9	20.0	15.0
300	30	43	3,932	0.9	22.5	15.0
300	75	43	1,572	0.9	21.4	15.0
300	150	43	786	0.9	20.0	15.0
300	300	36	324	0.9	100.0	15.0

## Abbreviations

The following abbreviations are used in this manuscript:

RP	Ring Polymer
LP	Linear Polymer
RLB	Ring-Linear Blend
NC	Non-Crossing
CX	Crossing

## References

[1] Roovers J., Toporowski P.M. (1988). Synthesis and characterization of ring polybutadienes. J. Polym. Sci. B.

[2] Bielawski C.W., Benitez D., Grubbs R.H. (2002). An “endless” route to cyclic polymers. Science.

[3] Endo K., Kobayashi S. (2008). Synthesis and properties of cyclic polymers. Advances in Polymer Science: New Frontiers in Polymer Synthesis.

[4] Tezuka Y. (2013). Topological Polymer Chemistry: Progress of Cyclic Polymers in Syntheses, Properties and Functions.

[5] Laib S., Robertson R.M., Smith D.E. (2006). Preparation and characterization of a set of linear DNA molecules for polymer physics and rheology studies. Macromolecules.

[6] Lee W., Lee H., Lee H.C., Cho D., Chang T., Gorbunov A.A., Roovers J. (2002). Retention behavior of linear and ring polystyrene at the chromatographic critical condition. Macromolecules.

[7] Takano A., Kushida Y., Aoki K., Masuoka K., Hayashida K., Cho D., Kawaguchi D., Matsushita Y. (2007). HPLC characterization of cyclization reaction product obtained by end-to-end ring closure reaction of a telechelic polystyrene. Macromolecules.

[8] Ohta Y., Kushida Y., Matsushita Y., Takano A. (2009). SEC–MALS characterization of cyclization reaction products: Formation of knotted ring polymer. Polymer.

[9] Ohta Y., Nakamura M., Matsushita Y., Takano A. (2012). Synthesis, separation and characterization of knotted ring polymers. Polymer.

[10] Robertson R.M., Laib S., Smith D.E. (2006). Diffusion of isolated DNA molecules: Dependence on length and topology. Proc. Natl. Acad. Sci. USA.

[11] Habuchi S., Satoh N., Yamamoto T., Tezuka Y., Vacha M. (2010). Multimode diffusion of ring polymer molecules revealed by a single-molecule study. Angew. Chem. Int. Ed..

[12] Habuchi S., Fujiwara S., Yamamoto T., Vacha M., Tezuka Y. (2013). Single-molecule study on polymer diffusion in a melt state: Effect of chain topology. Anal. Chem..

[13] Regan K., Ricketts S., Robertson-Anderson R.M. (2016). DNA as a model for probing polymer entanglements: Circular polymers and non-classical dynamics. Polymers.

[14] Fetters L.J., Lohse D.J., Graessley W.W. (1999). Chain dimensions and entanglement spacings in dense macromolecular systems. J. Polym. Sci. Polym. Phys. Ed..

[15] Kapnistos M., Lang M., Vlassopoulos D., Pyckhout-Hintzen W., Richter D., Cho D., Chang T., Rubinstein M. (2008). Unexpected power-law stress relaxation of entangled ring polymers. Nat. Mater..

[16] Robertson R.M., Smith D.E. (2007). Strong effects of molecular topology on diffusion of entangled DNA molecules. Proc. Natl. Acad. Sci. USA.

[17] Robertson R.M., Smith D.E. (2007). Self-diffusion of entangled linear and circular DNA molecules: Dependence on length and concentration. Macromolecules.

[18] Chapman C.D., Shanbhag S., Smith D.E., Robertson-Anderson R.M. (2012). Complex effects of molecular topology on diffusion in entangled biopolymer blends. Soft Matter.

[19] Gooßen S., Krutyeva M., Sharp M., Feoktystov A., Allgaier J., Pyckhout-Hintzen W., Wischnewski A., Richter D. (2015). Sensing polymer chain dynamics through ring topology: A neutron spin echo study. Phys. Rev. Lett..

[20] Papadopoulos G.D., Tsalikis D.G., Mavrantzas V.G. (2016). Microscopic dynamics and topology of polymer rings immersed in a host matrix of longer linear polymers: Results from a detailed molecular dynamics simulation study and comparison with experimental data. Polymers.

[21] McKenna G.B., Plazek D.J. (1986). The viscosity of blends of linear and cyclic molecules of similar molecular mass. Polym. Commun..

[22] Tead S.F., Kramer E.J., Hadziioannou G., Antonietti M., Sillescu H., Lutz P., Strazielle C. (1992). Polymer topology and diffusion—A comparison of diffusion in linear and cyclic macromolecules. Macromolecules.

[23] Mills P.J., Mayer J.W., Kramer E.J., Hadziioannou G., Lutz P., Strazielle C., Rempp P., Kovacs A.J. (1987). Diffusion of polymer rings in linear polymer matrices. Macromolecules.

[24] Vasquez R., Shanbhag S. (2011). Percolation of trace amounts of linear polymers in melts of cyclic polymers. Macromol. Theory Simul..

[25] Halverson J.D., Lee W.B., Grest G.S., Grosberg A.Y., Kremer K. (2011). Molecular dynamics simulation study of nonconcatenated ring polymers in a melt. II. Dynamics. J. Chem. Phys..

[26] Halverson J.D., Grest G.S., Grosberg A.Y., Kremer K. (2012). Rheology of ring polymer melts: From linear contaminants to ring-linear blends. Phys. Rev. Lett..

[27] Tsalikis D.G., Mavrantzas V.G. (2014). Threading of ring poly(ethylene oxide) molecules by linear chains in the melt. ACS Macro Lett..

[28] Tsalikis D.G., Koukoulas T., Mavrantzas V.G. (2014). Dynamic, conformational and topological properties of ring–linear poly(ethylene oxide) blends from molecular dynamics simulations. React. Funct. Polym..

[29] Lee E., Kim S., Jung Y. (2015). Slowing down of ring polymer diffusion caused by inter-ring threading. Macromol. Rapid Commun..

[30] Michieletto D., Marenduzzo D., Orlandini E., Alexander G.P., Turner M.S. (2014). Threading dynamics of ring polymers in a gel. ACS Macro Lett..

[31] Tsalikis D.G., Mavrantzas V.G., Vlassopoulos D. (2016). Analysis of slow modes in ring polymers: Threading of rings controls long-time relaxation. ACS Macro Lett..

[32] Yang Y.B., Sun Z.Y., Fu C.L., An L.J., Wang Z.G. (2010). Monte Carlo simulation of a single ring among linear chains: Structural and dynamic heterogeneity. J. Chem. Phys..

[33] Henke S.F., Shanbhag S. (2014). Self-diffusion in asymmetric ring-linear blends. React. Funct. Polym..

[34] Shanbhag S. (2016). Unusual dynamics of ring probes in linear matrices. J. Polym. Sci. B Polym. Phys..

[35] Graessley W.W. (1982). Entangled linear, branched and network polymer systems—Molecular theories. Adv. Polym. Sci..

[36] Klein J. (1986). Dynamics of entangled linear, branched, and cyclic polymers. Macromolecules.

[37] Hess W. (1987). Tracer diffusion in polymeric mixtures. Macromolecules.

[38] Kolinski A., Skolnick J., Yaris R. (1987). Monte Carlo studies on the long time dynamic properties of dense cubic lattice multichain systems. II. Probe polymer in a matrix of different degrees of polymerization. J. Chem. Phys..

[39] Barsky S. (2000). Molecular dynamics study of diffusion in bidisperse polymer melts. J. Chem. Phys..

[40] Lin H., Mattice W.L., von Meerwall E.D. (2007). Chain dynamics of bidisperse polyethylene melts: A Monte Carlo study on a high-coordination lattice. Macromolecules.

[41] Picu R.C., Rakshit A. (2007). Coarse grained model of diffusion in entangled bidisperse polymer melts. J. Chem. Phys..

[42] Wang Z., Larson R.G. (2008). Constraint release in entangled binary blends of linear polymers: A molecular dynamics study. Macromolecules.

[43] Green P.F., Mills P.J., Palmstrøm C.J., Mayer J.W., Kramer E.J. (1984). Limits of reptation in polymer melts. Phys. Rev. Lett..

[44] Green P.F., Kramer E.J. (1986). Matrix effects on the diffusion of long polymer chains. Macromolecules.

[45] Antonietti M., Coutandin J., Sillescu H. (1986). Diffusion of linear polystyrene molecules in matrixes of different molecular weights. Macromolecules.

[46] Seggern J.V., Klotz S., Cantow H.J. (1991). Reptation and constraint release in linear polymer melts: An experimental study. Macromolecules.

[47] Patteson A.E., Gopinath A., Purohit P.K., Arratia P.E. (2016). Particle diffusion in active fluids is non-monotonic in size. Soft Matter.

[48] Kasyap T.V., Koch D.L., Wu M. (2014). Hydrodynamic tracer diffusion in suspensions of swimming bacteria. Phys. Fluids.

[49] Weber S.N., Weber C.A., Frey E. (2016). Binary mixtures of particles with different diffusivities demix. Phys. Rev. Lett..

[50] Shaffer J.S. (1994). Effects of chain topology on polymer dynamics—Bulk melts. J. Chem. Phys..

[51] Iyer B.V.S., Lele A.K., Shanbhag S. (2007). What is the size of a ring polymer in a ring-linear blend?. Macromolecules.

[52] Iyer B.V.S., Shanbhag S., Juvekar V.A., Lele A.K. (2008). Self-diffusion coefficient of ring polymers in semidilute solution. J. Polym. Sci. B Polym. Phys..

[53] Subramanian G., Shanbhag S. (2008). Conformational properties of blends of cyclic and linear polymer melts. Phys. Rev. E.

[54] Subramanian G., Shanbhag S. (2009). Conformational free energy of melts of ring-linear polymer blends. Phys. Rev. E.

[55] Subramanian G., Shanbhag S. (2008). Self-diffusion in binary blends of cyclic and linear polymers. Macromolecules.

[56] Brown S., Lenczycki T., Szamel G. (2001). Influence of topological constraints on the statics and dynamics of ring polymers. Phys. Rev. E.

[57] Shanbhag S. (2013). Extraction of self-diffusivity in systems with nondiffusive short-time behavior. Phys. Rev. E.

[58] Shanbhag S. (2016). Estimating self-diffusion in polymer melts: How long is a long enough molecular simulation?. Mol. Simul..

[59] Shanbhag S., Larson R.G. (2005). Chain retraction potential in a fixed entanglement network. Phys. Rev. Lett..

[60] Shanbhag S., Larson R.G. (2006). Identification of topological constraints in entangled polymer melts using the bond-fluctuation model. Macromolecules.

[61] Uzcategui A.V., Shanbhag S. (2014). Self-entanglement of a single polymer chain confined in a cubic box. J. Polym. Sci. B Polym. Phys..

[62] Shaffer J.S. (1995). Effects of chain topology on polymer dynamics—Configurational relaxation in polymer melts. J. Chem. Phys..

[63] Tsolou G., Stratikis N., Baig C., Stephanou P.S., Mavrantzas V.G. (2010). Melt structure and dynamics of unentangled polyethylene rings: Rouse theory, atomistic molecular dynamics simulation, and comparison with the linear analogues. Macromolecules.

[64] Kuriata A., Sikorski A. (2015). Computer simulation of cyclic polymers in disordered media. Comput. Methods Sci. Technol..

